# Self-Inflicted Wrist Injuries in Flexor Tendon Zone V: Clinical Patterns, Management Strategies, and Outcomes

**DOI:** 10.7759/cureus.103658

**Published:** 2026-02-15

**Authors:** María F Hernández Pérez, Andrea Navalón Calzada, Aarón Gonzalez Espinosa, Alfonso Sandoval Polito, Colin A Ramirez Díaz, Emilio Mondragón Rosas, Dulce M Luquin López, Edson O Romero Cázares, José Emiliano González Flores

**Affiliations:** 1 Orthopedics and Traumatology, October 1st Regional High-Specialty Hospital, Institute for Social Security and Services for State Workers (ISSSTE), Mexico City, MEX; 2 Surgery, Spanish Hospital, Mexico City, MEX; 3 Plastic Surgery, General Hospital of Mexico "Dr. Eduardo Liceaga", Mexico City, MEX; 4 Medicine and Health Sciences, Tecnológico de Monterrey Campus Ciudad de Mexico, Mexico City, MEX; 5 Medicine, Meritorious Autonomous University of Puebla, Puebla City, MEX; 6 Surgery, University La Salle, Mexico City, MEX; 7 Medical Service, Mexican Army, Secretariat of National Defense, Mexico City, MEX

**Keywords:** flexor tendon injury, functional outcomes, hand trauma, neurovascular repair, patient-reported outcomes, rehabilitation protocols, self-inflicted wrist injury, spaghetti wrist, suicide attempt, zone v laceration

## Abstract

Self-inflicted wrist injuries, particularly those involving flexor tendon zone V, constitute a distinct and clinically challenging subset of upper-extremity trauma with profound surgical, functional, and psychosocial implications. For every suicide, many involve wrist lacerations capable of compromising tendinous, neural, and vascular structures and resulting in long-term disability. Despite their clinical relevance, the literature remains fragmented, with no universally accepted definition of “spaghetti wrist,” a term originally described in early surgical series of extensive volar wrist lacerations, and no standardized management framework. This narrative review critically synthesizes current evidence on epidemiology, injury mechanisms, classification systems, clinical assessment, management strategies, rehabilitation, complications, and outcomes. PubMed/MEDLINE, Scopus, and Google Scholar were searched for English- and Spanish-language studies published between January 2005 and January 2025, including cohort studies, case series, case reports, and reviews addressing self-inflicted injuries involving flexor tendon zone V. Extracted variables encompassed epidemiologic patterns, injury characteristics, classification systems, including the Verdan flexor tendon zone framework and heterogeneous “spaghetti wrist” definitions, diagnostic and outcome assessment tools, surgical and non-surgical management, rehabilitation protocols, and functional recovery. Findings were synthesized qualitatively to identify recurring patterns, areas of consensus, and persistent knowledge gaps. Available evidence indicates that these injuries predominantly affect young adults, frequently men, with a high prevalence of psychiatric comorbidity. Injury patterns characteristically involve the dominant hand acting upon the non-dominant wrist and demonstrate extensive multistructural compromise. Flexor tendons are almost universally injured, while neurovascular involvement varies but serves as the principal determinant of long-term functional prognosis. Substantial heterogeneity in reported outcomes reflects inconsistencies in injury definitions, surgical techniques, rehabilitation strategies, and evaluative metrics. Psychiatric factors further influence recurrence risk, adherence to therapy, and return-to-work trajectories. Self-inflicted flexor tendon zone V injuries, therefore, represent a complex and heterogeneous clinical entity. Although advances in microsurgical repair and structured rehabilitation have improved functional recovery, prognosis remains guarded in the presence of neurovascular damage. Standardized definitions, validated outcome measures, and integrated multidisciplinary care, encompassing surgical, rehabilitative, and psychiatric domains, are essential to enhance interstudy comparability and optimize patient-centered outcomes.

## Introduction and background

Self-inflicted wrist injuries may represent a distinct subset of trauma frequently encountered in emergency departments. It has been estimated that for every suicide, approximately 20 suicide attempts occur, and many of these involve wrist lacerations [1]. Such injuries can affect critical structures including tendons, nerves, and arteries, which may lead to long-term functional impairment [2]. Volar wrist lacerations, often referred to as “spaghetti wrist” or “full-house syndrome” [3], appear to carry a high risk of catastrophic sequelae [4]. Despite their clinical relevance, there is no universal agreement on the definition of spaghetti wrist; some authors describe it as the involvement of three structures (tendon, nerve, or artery), whereas others extend the definition to up to 10 structures [3,5]. In most reported cases, the dominant hand is used to injure the non-dominant hand [2].

Although wrist lacerations are relatively common, available literature on their classification, clinical assessment, and reported outcomes remains limited and fragmented. To date, no standardized integrated management protocols have been widely adopted for the treatment of self-inflicted flexor tendon zone V injuries, particularly those involving multistructural damage requiring coordinated surgical, rehabilitative, and psychiatric care. While structured surgical and rehabilitation protocols exist for flexor tendon injuries, their application to complex self-inflicted multistructural zone V trauma remains non-standardized. Assessment tools such as Noaman’s criteria [5,6] offer structured frameworks for evaluating tendon, nerve, and vascular involvement, though their complexity has limited their use in routine practice.

Recent multicenter data from England suggest that about 19.6% of all hospital presentations for self-harm involve cutting or stabbing, and of those, 69.4% are localized to the arm or wrist [7]. These findings highlight the frequency and potential importance of wrist injuries in the context of self-harm. Survivors often require complex surgical procedures and prolonged rehabilitation, which may result in considerable psychosocial and socioeconomic consequences [2,4]. Previous studies have primarily examined epidemiology [8] and functional recovery [9] of self-inflicted wrist injuries. However, to our knowledge, no comprehensive review has specifically focused on zone V injuries. This gap in the literature provides an opportunity for a narrative synthesis to summarize existing knowledge, explore areas of consensus and uncertainty, and contribute to a clearer understanding of these challenging injuries.

## Review

Methods

This study was designed as a narrative review aimed at consolidating and synthesizing the available literature on self-inflicted wrist injuries involving flexor tendon zone V. A narrative methodology was selected due to the marked heterogeneity of the existing evidence, which includes retrospective cohort studies, case series, case reports, and expert reviews. Such variability in study design, patient populations, and outcome reporting limits the feasibility of applying the structured framework required for a systematic review or meta-analysis.

Unlike systematic reviews that adhere to standardized reporting guidelines, narrative reviews allow for the integrative discussion of diverse forms of clinical and surgical evidence. This approach facilitates a comprehensive examination of epidemiology, injury patterns, anatomical considerations, management strategies, and functional outcomes, while also enabling the identification of conceptual gaps within the literature.

A literature search was conducted in PubMed/MEDLINE, Scopus, and Google Scholar for studies published between January 2005 and January 2025. Articles in English and Spanish were considered eligible. Included publication types comprised original research articles, retrospective and prospective cohort studies, case series, case reports, and review articles addressing self-inflicted wrist injuries, with particular emphasis on zone V involvement.

The search strategy incorporated Medical Subject Headings (MeSH) and free-text terms, including “self-inflicted wrist injury,” “zone V laceration,” “spaghetti wrist,” “full house syndrome,” “wrist cutting,” “volar forearm laceration,” and “flexor tendon repair zone V,” combined using Boolean operators (AND/OR). Reference lists of selected articles were manually screened to identify additional relevant publications.

Study selection was performed in two stages: title and abstract screening followed by full-text review. Articles not meeting inclusion criteria, duplicates, non-clinical publications, cadaveric or biomechanical studies without clinical outcomes, and reports focused exclusively on accidental injuries or anatomical zones outside zone V were excluded. When overlapping cohorts were identified, the most comprehensive study was retained.

**Figure 1:**
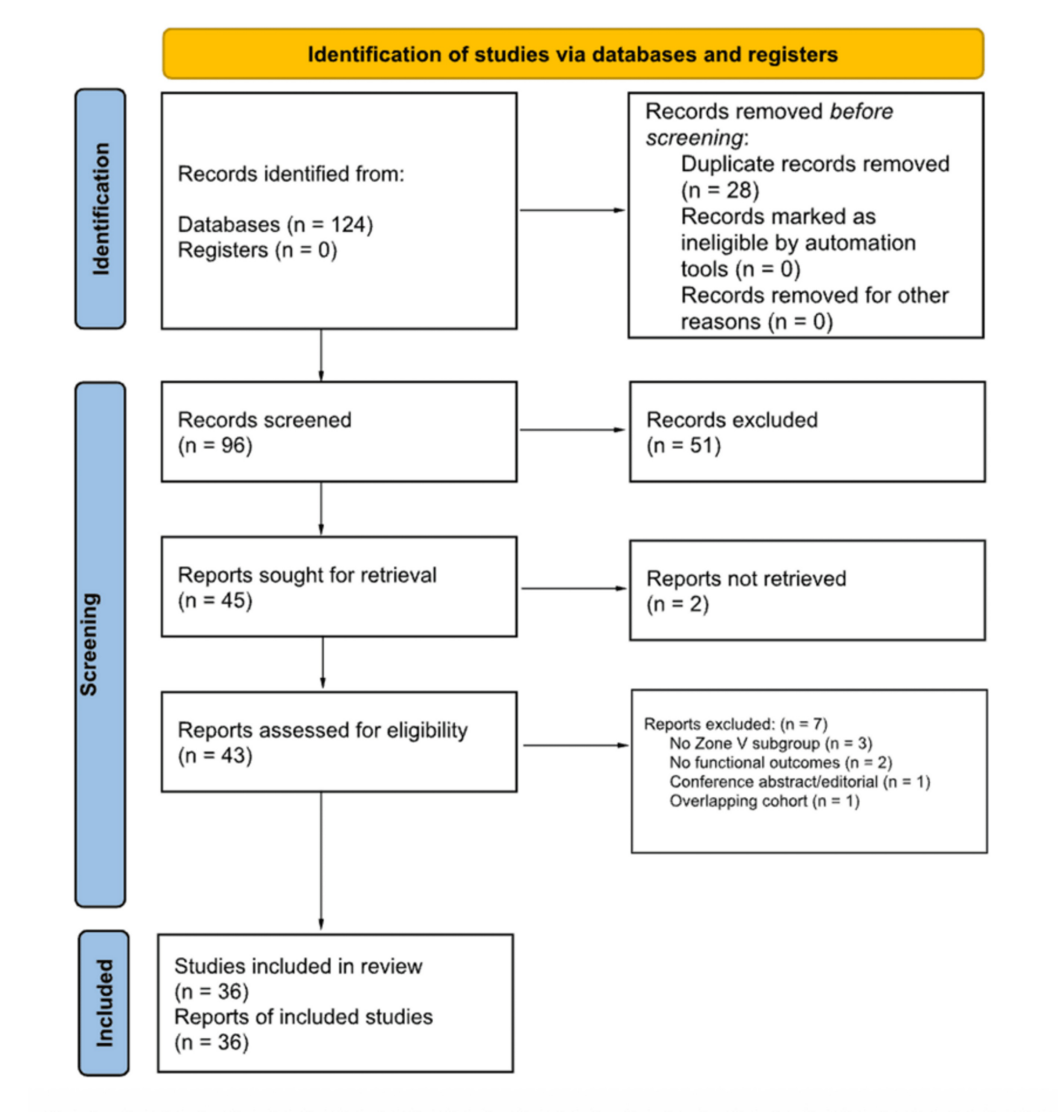
PRISMA-style flow diagram adapted for narrative review methodology The diagram outlines the structured literature selection process, including database identification, duplicate removal, title and abstract screening, full-text eligibility assessment, and final inclusion of studies addressing self-inflicted flexor tendon zone V wrist injuries. PRISMA: Preferred Reporting Items for Systematic Reviews and Meta-Analyses

The selection process is illustrated in a Preferred Reporting Items for Systematic Reviews and Meta-Analyses (PRISMA)-style flow diagram adapted for narrative review methodology (Figure 1). Although inter-rater agreement coefficients were not calculated, all inclusion decisions were made through reviewer consensus.

Data were extracted narratively, focusing on epidemiology, mechanisms of injury, classification systems, surgical management, rehabilitation protocols, and functional outcomes. When available, validated assessment tools such as Noaman’s criteria, Disabilities of the Arm, Shoulder, and Hand (DASH) score, and Total Active Motion (TAM) were recorded.

Given the heterogeneity of the included studies, no quantitative synthesis or meta-analysis was performed. Findings were instead qualitatively integrated to identify recurring clinical patterns and thematic consistencies.

Limitations of the Methodological Approach

As a narrative review, this study is inherently subject to methodological limitations, including potential selection bias, absence of standardized quality appraisal, and lack of statistical pooling. Additionally, variability in definitions (e.g., “spaghetti wrist”), outcome measures, and follow-up reporting across studies may influence the interpretability and generalizability of the findings.

Review

Epidemiology and Demographics

Self-inflicted wrist injuries represent a significant subset of self-harm presentations and have gained recognition as a clinically relevant trauma entity. Multicenter data from England indicate that approximately 19.6% of hospital presentations for self-harm involve cutting or stabbing mechanisms, of which 69.4% are localized to the arm or wrist [7]. This distribution highlights the wrist as a frequent anatomical target in self-inflicted injury, reflecting both its accessibility and its symbolic association with suicidal behavior. Notably, injury location has also been correlated with risk of subsequent suicide, suggesting prognostic implications beyond the immediate surgical context [7-9].

Contemporary surgical series further refine the epidemiological profile of patients sustaining zone V injuries. Wang et al. [10], in a cohort of 50 individuals with severe volar wrist and forearm lacerations, reported a predominance of young adult men. Injuries were most commonly inflicted using the dominant hand on the non-dominant wrist, a pattern consistently described in intentional self-harm. Psychiatric comorbidities were frequently documented, reinforcing the dual surgical and psychosocial dimensions of this patient population [10].

Demographic variables may also influence functional outcomes. In a systematic review, Cardoz Lobo et al. [6] observed that most patients with zone V flexor tendon injuries were between 20 and 40 years of age and often engaged in high-risk behaviors preceding injury. Although outcome reporting varied substantially across studies, both patient age and the number of injured structures were recurrently associated with prognosis [6].

Overall, available evidence indicates that self-inflicted zone V wrist injuries predominantly affect young adults, with a male predominance and a high prevalence of psychiatric comorbidity. Large epidemiological datasets provide population-level context, while surgical cohorts and systematic reviews offer more granular demographic characterization.

Mechanisms and Patterns of Injury

Self-inflicted wrist injuries demonstrate distinct mechanisms and anatomical patterns when compared with accidental trauma. Kisch et al. [2] reported that deep wrist lacerations associated with suicide attempts frequently involve multiple structures-including tendons, nerves, and arteries-whereas accidental injuries more commonly remain superficial. This distinction underscores the clinical importance of injury intentionality when assessing wound severity and planning surgical management.

In a multidisciplinary analysis of wrist lacerations, Jeong et al. [4] found that most self-inflicted injuries were inflicted with sharp instruments and predominantly affected the volar aspect of the non-dominant wrist, typically injured by the dominant hand. This recurrent pattern has been consistently observed across clinical series and reflects both accessibility and behavioral intentionality in self-harm mechanisms.

The extent of structural compromise in these injuries has historically been conceptualized under the term “spaghetti wrist.” Noaman [5] described this entity as simultaneous injury to tendons, nerves, and vascular structures at the volar wrist, frequently involving multiple anatomical components and necessitating complex reconstructive repair. Subsequent clinical series have reinforced the multistructural nature of these injuries. Ersen et al. [8], in a cohort of 41 patients with self-inflicted wrist lacerations, reported a high prevalence of combined tendon, nerve, and arterial involvement requiring urgent surgical intervention.

More recent studies have further refined anatomical characterization. Wang et al. [10] documented near-universal flexor tendon involvement in severe cases, with frequent concomitant injury to the median and ulnar nerves and the radial and ulnar arteries. Based on similar injury patterns and functional implications, the authors proposed extending the conceptual framework of “spaghetti wrist” to include proximal volar forearm lacerations.

Overall, current evidence indicates that self-inflicted wrist injuries most commonly involve the non-dominant wrist and are characterized by a high likelihood of multistructural damage. Flexor tendons are consistently affected, with variable degrees of neurovascular compromise depending on injury depth and mechanism.

Quantitative comparisons across cohorts further delineate these distinctions. While accidental lacerations more often involve isolated or superficial structures, self-inflicted injuries demonstrate multistructural involvement in the majority of cases, with flexor tendon injury approaching universal prevalence and neurovascular compromise reported in more than half of patients [2,4,5,8,11]. The dominant-to-non-dominant injury pattern has been documented in approximately 80%-85% of cases, with psychiatric comorbidities present in a substantial proportion of affected individuals [4,8,11].

Table 1 summarizes the principal clinical variables differentiating accidental from self-inflicted wrist injuries, highlighting recurrent mechanistic patterns and their surgical implications.

**Table 1:** Mechanisms and patterns of wrist injuries: accidental vs. self-inflicted (zone V focus)

Variable	Accidental injuries (e.g., occupational and domestic)	Self-inflicted injuries (suicidal/parasuicidal)	References
Intentionality	Single-event trauma without self-harm intent; multistructural involvement < 30%	Strong association with suicidal/parasuicidal behavior; multistructural involvement > 70%	[2]
Side/dominant hand involvement	No consistent pattern; mechanism-dependent	Predominantly non-dominant wrist injured by the dominant hand (80%–85%)	[4,8]
Instrument used	Machinery, tools, accidental sharp objects	Knives/blades (~64%), glass (~21%), household objects	[4]
Structures involved	Isolated tendon injuries more common; limited neurovascular compromise	Flexor tendons ~100%; median nerve 61%–82%; ulnar nerve 54%–65%; radial artery 49%–59%; ulnar artery 46%–53%	[5,8,11]
Number of injured structures (severity)	Usually ≤2 structures	≥3 structures in most cases; consistent with “spaghetti wrist” patterns	[5,11]
Anatomical location	Variable, mechanism-dependent	~94% located 1–3 cm proximal to wrist crease; transverse/oblique pattern	[11]
Age profile	Heterogeneous; includes older adults	Mean age 29–32 years; young adults (20–40 yrs) most affected; male predominance 73%–76%	[8,11]
Psychiatric comorbidity	Rarely documented	Present in ~68%; depression and borderline personality disorder most frequent	[4,8]
Expected functional pattern	Limited functional deficit; generally favorable prognosis	Severe motor impairment, sensory deficits, ischemia risk	[2,5,11]

Definitions and Classification Systems

The terminology and classification of complex volar wrist injuries remain inconsistent, reflecting ongoing debate within the literature. The term “spaghetti wrist” was originally introduced to describe extensive volar lacerations involving multiple structures, with exposed tendon ends resembling strands of spaghetti [5]. Despite its widespread use, no universal consensus exists regarding the minimum number or type of structures required to fulfill this definition. Some authors consider injury to three structures sufficient, typically involving tendons, nerves, and vessels, whereas others propose broader thresholds extending to as many as 10 injured components [5,11].

**Figure 2:**
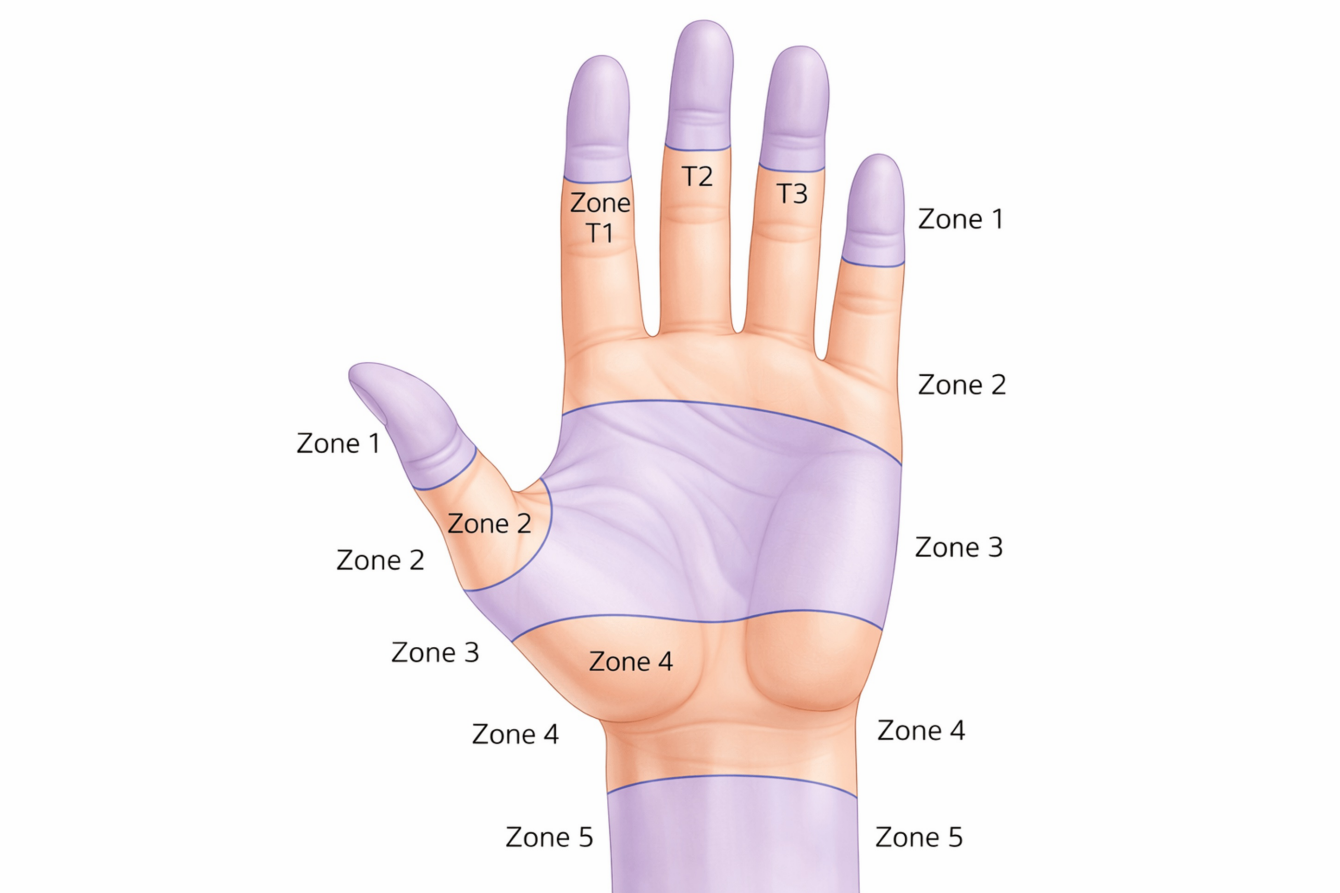
Flexor tendon zonal anatomy of the hand and wrist (palmar view) This original illustration depicts the palmar surface of the hand and distal forearm, highlighting the anatomical distribution of the flexor tendon zones according to the Verdan classification. The diagram delineates zones I–V along the fingers, palm, and wrist, as well as the terminal digital subzones (T1–T3) at the fingertip level. Curved demarcation lines indicate the anatomical boundaries of each zone. Zone I extends from the fingertip to the insertion of the flexor digitorum superficialis (FDS) tendon. Zone II spans from the FDS insertion to the distal palmar crease, representing the region traditionally associated with complex tendon repair. Zone III covers the palm, zone IV corresponds to the carpal tunnel region, and zone V extends proximally to the distal forearm. This zonal classification is clinically relevant for surgical planning, prognostic assessment, and rehabilitation strategies in flexor tendon and volar wrist injuries. The figure is original and was created by the authors.

**Figure 3:**
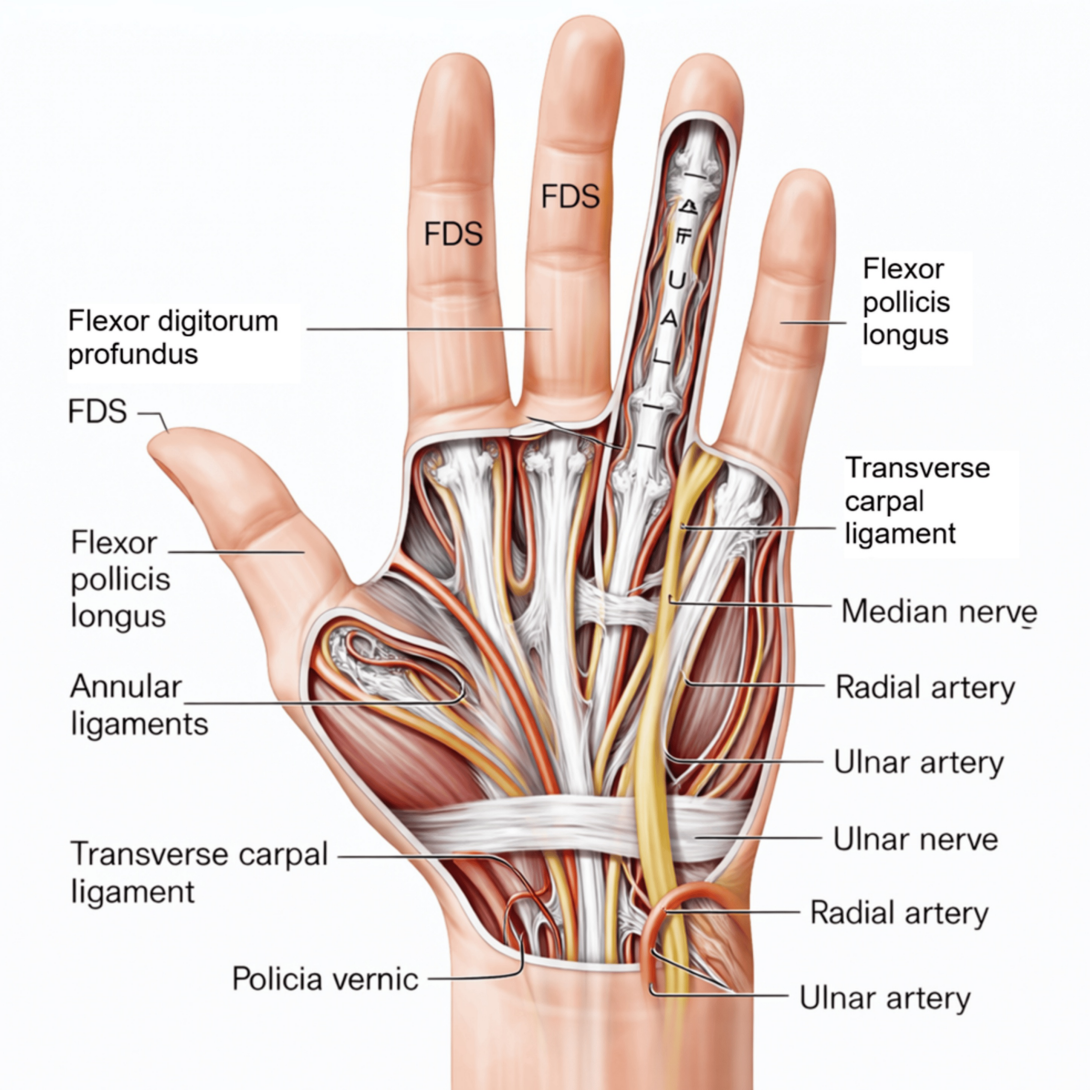
Palmar anatomy of flexor tendons, neurovascular structures, and transverse carpal ligament This original illustration depicts the palmar anatomy of the hand and distal wrist, highlighting the spatial arrangement of flexor tendons, major neurovascular bundles, and key ligamentous structures relevant to volar wrist injuries. The flexor digitorum superficialis (FDS) and flexor digitorum profundus tendons are shown along the digital rays, while the flexor pollicis longus tendon is illustrated within the thumb compartment. The transverse carpal ligament is represented spanning the carpal tunnel, beneath which the median nerve is visualized in close relationship to the flexor tendons. The ulnar nerve and artery are depicted within Guyon’s canal, while the radial artery is illustrated along the radial aspect of the distal wrist. This anatomical configuration underscores the high risk of combined tendinous, neural, and vascular injury in deep volar lacerations, particularly those involving flexor tendon zone V. The figure is original and was created by the authors.

Figures 2, 3 illustrate the anatomical framework underlying this classification challenge. The close spatial relationship between flexor tendons, the median and ulnar nerves, and the radial and ulnar arteries within the volar wrist and distal forearm explains the high likelihood of multistructural involvement in deep lacerations and supports the conceptual grouping of these injuries under a unified descriptive term.

Beyond structural definitions, several frameworks have been proposed to standardize outcome reporting. Noaman’s criteria remain the most frequently cited system, incorporating tendon recovery, motor function of the median and ulnar nerves, sensory assessment, deformity, and range of motion [5]. Although comprehensive, its complexity has limited routine clinical adoption. Subsequent systematic reviews have highlighted substantial heterogeneity in functional evaluation. Cardoz Lobo et al. [6] identified at least seven distinct outcome measures used across studies of zone V flexor tendon injuries, including TAM and DASH, underscoring the absence of a standardized reporting framework.

More recent contributions have sought to refine existing conceptual boundaries. Wang et al. [10] proposed extending the definition of “spaghetti wrist” proximally to include severe volar forearm lacerations with comparable multistructural patterns and functional consequences. Similarly, Kim et al. [11] documented cases involving up to 10 injured structures, further challenging narrower definitional thresholds. A contemporary review by Rodica et al. [12] contextualized the historical evolution of the term and concluded that a unified classification system has yet to be established.

Clinical series reinforce the practical implications of this variability. Ersen et al. [8], in a retrospective cohort of self-inflicted wrist injuries, reported a high prevalence of multistructural involvement accompanied by heterogeneous functional outcomes, reflecting the limitations of existing classification and assessment models.

Overall, current literature demonstrates substantial variability in both structural definitions and outcome reporting systems for complex volar wrist injuries. Table 2 summarizes the principal classification approaches and definitional thresholds described to date, highlighting areas of convergence as well as persistent limitations.

**Table 2:** Definitions and classification systems of “spaghetti wrist” injuries ROM: range of motion

Author/source	Year	Proposed definition	Key features	Limitations
Noaman [5]	2007	≥3 structures (tendons, nerves, vessels) injured at the wrist	Established classic definition; introduced functional outcome criteria (tendon, motor, deformity, sensory, ROM)	Arbitrary structural threshold; time-consuming assessment tool; limited widespread adoption
Ersen et al. [8]	2017	Multistructural volar wrist lacerations resulting from self-inflicted wrist cutting	Retrospective cohort of 41 cases; high prevalence of multistructural injury; urgent surgical repair frequently required; heterogeneous functional outcomes	Retrospective design; single-center cohort; non-standardized outcome reporting
Cardoz Lobo et al. [6]	2023	Emphasized heterogeneity in outcome definitions; ≥7 assessment tools used in zone V injuries	Systematic review; highlighted variability in outcome reporting; underscored need for standardization	Did not propose a structural definition; focused on outcome frameworks
Wang et al. [10]	2022	Proposed extending “spaghetti wrist” to proximal volar forearm lacerations	Recognized comparable multistructural patterns and functional impact beyond wrist crease	Expansion not universally accepted; derived from limited cohort data
Kim et al. [11]	2021	Up to 10 structures considered within the definition of spaghetti wrist	Detailed anatomical characterization; high prevalence of tendon and nerve injury	Small sample size; limited long-term functional outcomes
Rodica et al. [12]	2025	Narrative synthesis of historical and modern definitions; no unified consensus identified	Integrated surgical, rehabilitative, and diagnostic perspectives; emphasized definitional evolution	Did not establish definitional thresholds; highlighted need for consensus

Clinical Assessment and Diagnostic Tools

Accurate assessment of self-inflicted wrist injuries in zone V is essential for surgical planning and prognostic estimation. Initial evaluation relies on a comprehensive neurovascular examination, including capillary refill, distal pulse assessment, and Allen’s test when vascular compromise is suspected. Flexor tendon integrity is assessed through isolated finger flexion testing, while sensory function is evaluated using light touch and two-point discrimination within the median and ulnar nerve distributions. Despite their fundamental role, bedside examinations may be limited in the acute setting by pain, edema, and reduced patient cooperation [5,8].

Beyond initial clinical assessment, several frameworks have been developed to standardize postoperative functional evaluation. Noaman’s criteria remain the most frequently cited system, integrating tendon recovery, motor and sensory nerve function, deformity, and range of motion into a composite outcome measure [5]. However, its complexity has limited widespread clinical implementation. More pragmatic instruments-such as TAM and the DASH score-are commonly employed, though each evaluates distinct functional domains, which may limit cross-study comparability [6].

Contemporary literature increasingly emphasizes the importance of systematically documenting neurovascular involvement during assessment. Cohort analyses have demonstrated that the extent of associated nerve injury substantially influences functional recovery and long-term disability, reinforcing the need for integrated tendon and nerve evaluation in zone V trauma [13,14].

Overall, while structured bedside examination remains indispensable, variability in functional assessment tools continues to limit standardization of outcome reporting. Table 3 summarizes the principal diagnostic and evaluative instruments used in the assessment of complex volar wrist injuries, outlining their respective strengths and limitations.

**Table 3:** Commonly used outcome measures in spaghetti wrist and zone V injuries

Outcome measure	Components assessed	Advantages	Limitations	References
Noaman’s criteria	Tendon function; motor recovery of median and ulnar nerves; sensory outcomes; deformity; range of motion	Comprehensive; injury-specific; integrates multistructural assessment	Time-consuming; limited adoption; not externally validated	[5,8]
TAM (Total Active Motion)	Composite arc of finger flexion and extension	Simple; widely used in tendon literature; easy to calculate	Focuses on mobility; does not assess sensory or vascular recovery	[6,14]
DASH (Disabilities of the Arm, Shoulder, and Hand)	30-item patient-reported disability questionnaire	Patient-centered; validated; captures functional impact and quality of life	Non-specific to wrist trauma; may underrepresent isolated tendon or nerve deficits	[6,14]
MRC (Medical Research Council) scale	Grading of muscle strength (0–5)	Reproducible; widely accepted for nerve recovery assessment	Limited to motor function; does not evaluate tendon integrity or sensory outcomes	[13]
Sensory tests (2-point discrimination, Semmes–Weinstein monofilament)	Sensory recovery in median and ulnar nerve territories	Quick; clinically informative; bedside applicable	Examiner variability; subjective thresholds; limited standardization	[8,13]

Management Strategies

The management of self-inflicted wrist injuries in zone V presents unique challenges due to their frequent multistructural involvement and the psychosocial context in which they occur. Core surgical principles include early operative exploration; meticulous repair of injured tendons, nerves, and vascular structures; and coordinated postoperative rehabilitation. Despite general agreement on these principles, considerable variability persists in operative techniques and perioperative strategies.

Flexor tendon repair is typically performed using multistrand core suture techniques, commonly reinforced with epitendinous sutures to enhance tensile strength and facilitate tendon gliding [5,8]. Concomitant neurovascular injuries are addressed during the same operative setting. Median and ulnar nerve lacerations are most often managed with epineural repair, while arterial injuries involving the radial or ulnar arteries may require primary end-to-end anastomosis or interposition vein grafting when tension-free repair is not feasible [10,11].

The timing of surgical intervention remains an area of clinical debate. While early repair-often within 24 hours-is widely advocated to optimize structural and functional outcomes, delayed reconstruction may be considered in the presence of severe contamination, hemodynamic instability, or unresolved psychiatric risk requiring staged management [6,12].

Postoperative mobilization strategies vary substantially. Early controlled mobilization has been associated with improved tendon gliding and reduced adhesion formation but may carry an increased risk of repair rupture in complex multistructural injuries. Conversely, prolonged immobilization may protect delicate repairs while predisposing patients to stiffness and delayed functional recovery [6,14,15]. Meta-analytic evidence supports the functional benefits of early mobilization, although optimal timing and protocol intensity remain heterogeneous across studies [16].

Longitudinal cohort data increasingly emphasize the importance of patient-reported outcomes alongside objective functional measures. Improvements in grip strength, active motion, and perceived functional independence have been documented following structured tendon repair and rehabilitation programs [15].

Multidisciplinary care represents a critical component of comprehensive management. Integration of psychiatric evaluation and psychological support is particularly relevant in self-inflicted injuries, where surgical recovery is closely intertwined with psychosocial stabilization and long-term reintegration [12].

Overall, although fundamental surgical principles are broadly established, no universally accepted management algorithm exists for self-inflicted zone V wrist injuries. Variability in operative timing, reconstructive technique, and rehabilitation protocols reflects both the anatomical complexity of these injuries and the limited availability of high-level comparative evidence.

Table 4 summarizes the principal surgical and rehabilitative strategies described in the literature, highlighting areas of consensus and ongoing debate.

**Table 4:** Management strategies for self-inflicted zone V wrist injuries (“spaghetti wrist”) ROM: range of motion; DASH: Disabilities of the Arm, Shoulder, and Hand; TAM: Total Active Motion

Variable	Consensus/common practice	Variability/debate	References
Timing of repair	Early repair (<24 h) recommended to optimize outcomes	Delayed repair in contaminated wounds, unstable psychiatric status, or staged procedures	[6,12]
Tendon repair technique	Multistrand core sutures (4–6 strands) with epitendinous augmentation	Suture configuration and material vary; limited comparative trials	[5,8,13]
Nerve repair	Primary epineural end-to-end repair preferred; microsurgical techniques standard	Role of nerve grafts or conduits debated; outcomes depend on tension and gap length	[8,10,13]
Vascular repair	End-to-end anastomosis for radial/ulnar arteries; vein grafts for segmental loss	Need for repair when one artery remains intact remains debated	[10,11]
Soft-tissue coverage	Primary closure when feasible; local/regional flaps for exposed structures	Flap selection varies by defect size and location; free flaps rarely required	[12]
Immobilization vs. early mobilization	Early protected mobilization reduces adhesions and stiffness	Higher rupture risk in multistructural repairs; some centers favor prolonged immobilization	[6,14,16]
Rehabilitation protocols	Structured hand therapy essential; early ROM improves tendon gliding	Lack of standardization in timing, frequency, and intensity	[6,16]
Outcome measures	Noaman’s criteria, TAM, and DASH commonly utilized	Heterogeneity persists; patient-reported outcomes underutilized	[6,14,15]
Predictors of prognosis	Extent of nerve injury influences functional recovery	Psychiatric comorbidity and delayed rehabilitation worsen outcomes	[8,13,15]
Complications	Adhesions, stiffness, sensory deficits, ischemia	Re-rupture risk ~5%–15% depending on mobilization protocol	[8,13,16]
Psychiatric integration	Psychiatric evaluation recommended in all self-inflicted cases	Implementation inconsistent across centers	[12]

Rehabilitation and Functional Outcomes

Rehabilitation represents a critical determinant of recovery following self-inflicted zone V wrist injuries, as surgical repair alone does not guarantee functional restoration. Contemporary protocols emphasize balancing protection of multistructural repairs with early mobilization to minimize adhesions and joint stiffness. However, substantial variability persists regarding the optimal rehabilitation regimen, reflecting differences in institutional practices and injury severity [6,14].

Early controlled mobilization has consistently demonstrated benefits in tendon gliding and total active motion. In a systematic review and meta-analysis, Xu et al. [16] reported a superior range of motion and functional scores among patients undergoing early mobilization protocols, although these approaches may carry an increased risk of repair rupture in complex injuries. Conversely, prolonged immobilization may reduce rupture risk but is frequently associated with stiffness and delayed functional recovery, particularly in cases involving combined tendon and nerve injury [14].

Reported functional outcomes remain heterogeneous. Clinical series have documented satisfactory tendon recovery but incomplete sensory restoration, reflecting the biological constraints of nerve regeneration [8]. Prospective cohort data further illustrate this variability. Rosales et al. [17] reported that 73% of repaired tendons achieved “excellent” or “good” functional outcomes at the 12-month follow-up, accompanied by improvements in grip strength and patient-reported functional independence. Complementary evidence from systematic reviews underscores the importance of structured rehabilitation protocols while highlighting inconsistencies in therapy design, duration, and outcome measurement [18].

Neurovascular involvement remains an important modifier of recovery trajectories. Large epidemiological analyses have demonstrated the long-term functional and socioeconomic burden associated with upper-extremity nerve injuries, reinforcing the need for integrated tendon and nerve rehabilitation strategies and standardized outcome reporting [14].

Overall, rehabilitation following self-inflicted zone V wrist injuries requires an individualized, multidisciplinary approach that integrates early mobilization principles, protection of complex repairs, and validated functional and patient-reported outcome measures. Persistent heterogeneity in rehabilitation protocols and outcome frameworks continues to limit cross-study comparability and represents a priority area for future research.

Table 5 summarizes key clinical series and systematic reviews evaluating functional outcomes after surgical repair of zone V wrist injuries, including study design, follow-up duration, outcome measures, and principal findings.

**Table 5:** Reported functional outcomes after surgical repair of zone V wrist injuries Summary of representative clinical series and systematic reviews evaluating postoperative functional recovery, including objective functional metrics, PROs, and rehabilitation-associated findings. DASH: Disabilities of the Arm, Shoulder and Hand; N/A: not applicable; NR: not reported; ROM: range of motion; TAM: Total Active Motion

Study/author	Year	Sample size	Follow-up	Main outcomes measured	Key findings
Ersen et al. [8]	2017	41 patients	NR	Injury patterns; need for surgical intervention; complications	High prevalence of multistructural injuries following self-inflicted wrist cutting; urgent surgical repair frequently required; heterogeneous functional outcomes
Bircan et al. [13]	2005	42 patients	Mean 31 months	Functional outcomes; ROM; grip strength	Functional recovery influenced by injury severity; poorer outcomes observed in cases with associated neurovascular involvement
Rosales et al. [15]	2025	45 patients (65 tendons)	12 months	Grip strength; ROM; patient-reported outcomes (PROs)	Significant improvements in ROM and grip strength; enhanced patient-reported functional independence at follow-up
Xu et al. [16]	2023	Systematic review and meta-analysis (12 studies)	N/A	ROM; functional scores (TAM, DASH)	Early mobilization associated with improved functional outcomes, though rupture risk modestly increased
Williams et al. [17]	2025	38 patients	12 months	TAM; grip strength; DASH; PROs	Early active mobilization improved ROM and grip strength; PROs revealed residual disability not fully captured by clinical metrics
Peters et al. [18]	2021	Systematic review (Cochrane)	N/A	Rehabilitation protocols; functional recovery; complications	Structured rehabilitation associated with improved outcomes; variability in therapy regimens and outcome reporting limits comparability

Complications and Prognosis

Complications following zone V wrist injuries are common and represent a major determinant of long-term functional prognosis. The most frequently reported adverse outcomes include tendon adhesions, joint stiffness, and persistent sensory deficits, while tendon rupture and vascular compromise occur less commonly [8,13]. Reported complication rates vary substantially across studies, reflecting heterogeneity in injury severity, surgical technique, and postoperative rehabilitation protocols.

Large cohort analyses have helped clarify predictors of adverse recovery [19]. In a retrospective series of 1,031 patients, Jia et al. [20] identified complex multistructural injury patterns, delayed rehabilitation, and associated nerve damage as independent risk factors for tendon adhesions-the most prevalent cause of long-term functional limitation. Similarly, Tessier et al. [19] reported that rupture, infection, and reoperation rates after flexor tendon repair remain clinically significant even in high-volume centers, underscoring the ongoing challenge of optimizing repair durability.

Neurovascular involvement remains one of the most consistent prognostic modifiers. Bircan et al. [13] demonstrated that functional recovery after zone V flexor tendon repair was significantly poorer in the presence of concomitant nerve injury, with persistent motor and sensory deficits despite satisfactory tendon healing. Complementary findings from Lad et al. [21] further support this observation, showing superior outcomes in tendon-only injuries compared with combined tendon-nerve trauma, highlighting the prognostic weight of neurovascular lesions.

Overall, although advances in microsurgical repair and structured rehabilitation have improved functional outcomes, complication rates remain substantial and prognosis variable. Persistent heterogeneity in outcome reporting and follow-up methodologies limits cross-study comparability and reinforces the need for prospective multicenter investigations employing standardized functional assessment frameworks.

Discussion

This narrative review synthesizes current evidence on self-inflicted zone V wrist injuries-commonly referred to as “spaghetti wrist”-highlighting the complexity of their clinical management and the persistent lack of standardization across diagnostic, surgical, and rehabilitative domains. Despite advances in microsurgical techniques and structured rehabilitation protocols, functional outcomes remain variable, reflecting differences in injury severity, institutional practice patterns, and methodological heterogeneity within the literature.

Definitions and Conceptual Challenges

One of the most significant barriers to comparability across studies remains the absence of a universally accepted definition of “spaghetti wrist.” Reported thresholds for structural involvement vary widely, ranging from injury to three anatomical components to extensive multistructural compromise exceeding 10 structures [5,11]. As Tang [22] observed, definitional breadth directly influences cohort composition and outcome interpretation, with stricter inclusion criteria often associated with higher reported complication rates. Historical surgical series further underscore this variability, reflecting evolving conceptual frameworks and inconsistent injury classification. Collectively, this lack of definitional consensus continues to hinder meaningful cross-study comparisons and the development of standardized reporting frameworks.

Surgical Management Considerations

Advances in microsurgical repair have substantially improved the technical feasibility of reconstructing multistructural volar wrist injuries. However, biological limitations-particularly related to nerve regeneration and adhesion formation-continue to constrain functional recovery [23]. Early clinical series demonstrated that even technically successful repair of tendons and neurovascular structures does not uniformly translate into full functional restoration, with residual stiffness and sensory deficits frequently reported [24,25]. Contemporary practice has benefited from improved suture techniques, magnification, and perioperative care, yet outcome variability persists, reflecting the intrinsic severity of these injuries rather than purely technical limitations.

Rehabilitation and Functional Recovery

Postoperative rehabilitation remains a central determinant of outcome but also one of the most heterogeneous domains of care. Evidence consistently supports early controlled mobilization as a strategy to improve tendon gliding and range of motion, although this must be balanced against the risk of repair rupture in complex multistructural injuries [26-30]. Systematic reviews and comparative cohort studies have demonstrated superior functional recovery with early mobilization protocols, yet variability in therapy design, timing, and intensity continues to limit consensus regarding optimal rehabilitation strategies [30,31]. Injury severity and structural involvement further influence rehabilitation requirements, reinforcing the need for individualized therapy planning.

Prognostic Determinants

Long-term outcomes following spaghetti wrist injuries are influenced by multiple interrelated factors, including structural injury burden, timeliness of repair, and adherence to rehabilitation. Among these, neurovascular involvement consistently emerges as one of the most significant prognostic modifiers. Longitudinal cohort analyses demonstrate that patients with combined tendon and nerve injuries experience lower functional recovery, persistent sensory deficits, and delayed return to work compared with tendon-only injuries [32-34]. These findings highlight the compounded biological and functional impact of multistructural trauma.

Psychiatric and Multidisciplinary Dimensions

Given the intentional nature of these injuries, psychosocial factors represent an essential yet frequently underreported component of care. Retrospective analyses of wrist-cutting suicide attempts demonstrate a high prevalence of underlying psychiatric vulnerability and recurrent self-harm behavior [29]. Functional recovery is therefore influenced not only by surgical and rehabilitative variables but also by psychological status, treatment adherence, and social reintegration. Multidisciplinary care models integrating psychiatric evaluation, surgical reconstruction, and structured rehabilitation have been proposed as essential frameworks for optimizing both functional and psychosocial outcomes [35,36].

Knowledge Gaps and Future Directions

Despite decades of clinical experience, the evidence base surrounding self-inflicted zone V wrist injuries remains limited by methodological heterogeneity. Most available studies are retrospective, single-center analyses with inconsistent inclusion criteria and outcome reporting systems. Rehabilitation protocols lack standardization, and functional assessment tools vary widely, complicating comparative analysis. Future research should prioritize multicenter prospective cohorts, unified injury definitions, standardized rehabilitation algorithms, and integration of validated patient-reported outcome measures. Establishing such frameworks will be essential to improving comparability, guiding evidence-based management, and optimizing long-term recovery in this complex patient population.

## Conclusions

Self-inflicted wrist injuries involving flexor tendon zone V-classically termed “spaghetti wrist”-constitute one of the most surgically and functionally demanding patterns of upper-extremity trauma. Their clinical complexity derives from the frequent convergence of multistructural damage, including tendinous, neural, and vascular components, compounded by the psychosocial context in which these injuries occur. Despite advances in microsurgical techniques and structured rehabilitation pathways, outcome variability remains substantial, reflecting persistent heterogeneity in definitions, management strategies, and functional assessment frameworks. Neurological involvement continues to represent the principal determinant of long-term disability, often outweighing isolated tendon injury in prognostic relevance. Equally, psychiatric comorbidity, social vulnerability, and barriers to rehabilitation adherence critically shape recovery trajectories, reintegration capacity, and recurrence risk-underscoring that functional restoration extends beyond technical surgical success.

Moving forward, progress in this field will depend on the development of standardized injury classifications, harmonized outcome reporting, and prospective multicenter collaboration. The integration of patient-reported outcomes, functional performance metrics, and psychosocial variables will be essential to generate clinically translatable evidence. Ultimately, optimizing care for patients with self-inflicted zone V wrist injuries demands a multidisciplinary paradigm that aligns surgical expertise, rehabilitative science, and mental health intervention within a unified, patient-centered framework.
